# The cobalt(II) salt of the azo dye Orange G

**DOI:** 10.1107/S1600536810037360

**Published:** 2010-09-30

**Authors:** Alan R. Kennedy, Scott C. McKellar, Maurice O. Okoth

**Affiliations:** a Department of Pure & Applied Chemistry, University of Strathclyde, 295 Cathedral Street, Glasgow G1 1XL, Scotland; b Department of Chemistry and Biochemistry, Moi University, PO Box 1125-30100, Eldoret, Kenya.

## Abstract

Crystallizing the cobalt(II) salt of the azo dye Orange G from water was found to give the solvent-separated ion-pair species hexa­aqua­cobalt(II) 7-oxo-8-(2-phenyl­hydrazin-1-ylidene)-7,8-dihydro­naphthalene-1,3-disulfonate tetra­hydrate, [Co(H_2_O)_6_](C_16_H_10_N_2_O_7_S_2_)·4H_2_O. The asymmetric unit of the cobalt(II) salt contains three independent octa­hedral [Co(OH_2_)_6_]^2+^ cations, three azo anions, all with similar configurations, and 12 uncoordinated water mol­ecules. The structure is closely related to that of one of the known magnesium analogues. Both structures have *Z*′ = 3, feature nearly planar azo anions [maximum displacement of azo-N atoms from the plane of the phenyl ring = 0.058 (7) Å] in their hydrazone tautomeric form, form layer structures with hydro­philic and hydro­phobic layers alternating along the *b*-axis direction, and are stabilized by an extensive network of hydrogen bonds..

## Related literature

For other structures of metal salts of Orange G, see: Ojala *et al.* (1994 ▶); Kennedy *et al.* (2006 ▶). For the structural classification of metal salts of other sulfonated azo dyes and pigments, see: Kennedy *et al.* (2001 ▶, 2009 ▶). For the general coordination behaviour of —*R*SO_3_ species with metals, see: Côté & Shimizu (2003 ▶).
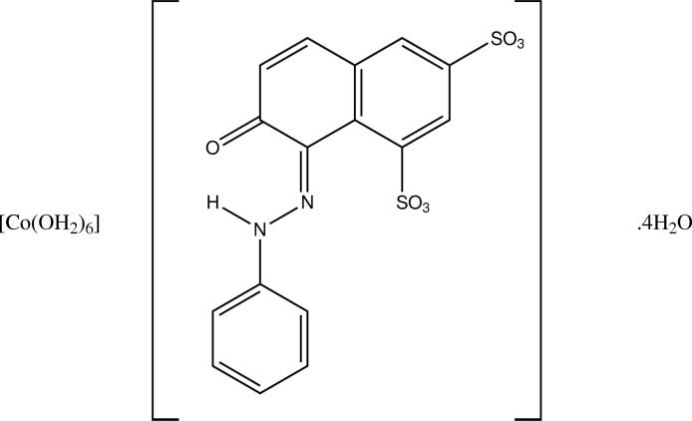


## Experimental

### 

#### Crystal data

[Co(H_2_O)_6_](C_16_H_10_N_2_O_7_S_2_)·4H_2_O
*M* *_r_* = 645.47
Triclinic, 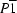

*a* = 10.0768 (3) Å
*b* = 14.6463 (5) Å
*c* = 26.3881 (8) Å
α = 93.470 (2)°
β = 90.813 (1)°
γ = 101.175 (1)°
*V* = 3812.4 (2) Å^3^
*Z* = 6
Mo *K*α radiation
μ = 0.92 mm^−1^
*T* = 123 K
0.35 × 0.22 × 0.06 mm

#### Data collection

Nonius KappaCCD diffractometer
Absorption correction: multi-scan (*SADABS*; Sheldrick, 2000 ▶) *T*_min_ = 0.683, *T*_max_ = 1.000
63505 measured reflections
16529 independent reflections
10980 reflections with *I* > 2σ(*I*)
*R*_int_ = 0.086

#### Refinement

*R*[*F* ^2^ > 2σ(*F* ^2^)] = 0.056
*wR*(*F* ^2^) = 0.150
*S* = 1.03
16529 reflections
1216 parameters
90 restraints
H atoms treated by a mixture of independent and constrained refinement
Δρ_max_ = 0.95 e Å^−3^
Δρ_min_ = −1.03 e Å^−3^

### 

Data collection: *COLLECT* (Hooft, 1988 ▶) and *DENZO* (Otwin­owski & Minor, 1997 ▶); cell refinement: *COLLECT* and *DENZO*; data reduction: *DENZO* (Otwinowski & Minor, 1997 ▶); program(s) used to solve structure: *SIR2004* (Burla *et al.*, 2005 ▶); program(s) used to refine structure: *SHELXL97* (Sheldrick, 2008 ▶); molecular graphics: *ORTEP-3* (Farrugia, 1997 ▶); software used to prepare material for publication: *SHELXL97*.

## Supplementary Material

Crystal structure: contains datablocks global, I. DOI: 10.1107/S1600536810037360/wm2388sup1.cif

Structure factors: contains datablocks I. DOI: 10.1107/S1600536810037360/wm2388Isup2.hkl

Additional supplementary materials:  crystallographic information; 3D view; checkCIF report

## Figures and Tables

**Fig. 1.**
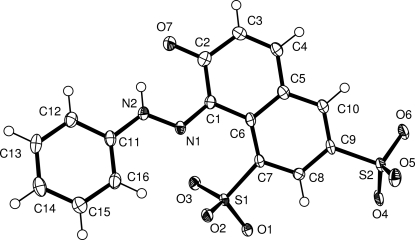
The molecular structure of one of the unique anions of Orange G, showing 50% probability displacement ellipsoids.

**Fig. 2.**
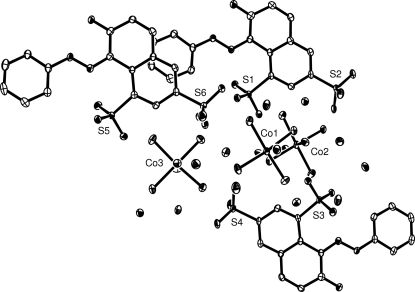
Contents of the asymmetric unit with 50% probability displacement ellipsoids. Hydrogen atoms are omitted for clarity.

**Fig. 3.**
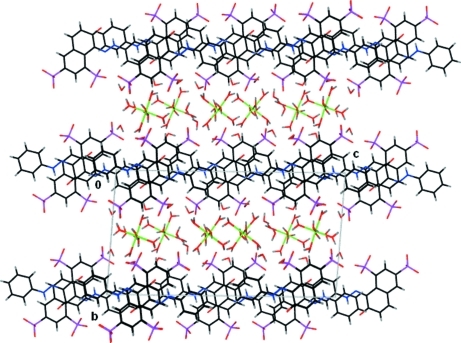
Packing diagram with view along the a axis. Note the alternate hyrophobic and hydrophilic layers.

## supplementary crystallographic information

### Comment

It is known that transition metal complexes of sulfonated aryl species generally form solvent-separated ion-pairs, but that *s*-block metals may form direct *M*—O_3_S bonds (Côté & Shimizu, 2003). A subgroup of these compounds, sulfonated azo molecules, are widely used as colourants. Despite this, there are relatively few known crystal structures of sulfonated azo colourants (Kennedy *et al.*, 2001). This is at least partly due to the fact that their salt forms typically give poor quality crystals with extremely anisotropic habits. Recently, a systematic structural study of *s*-block metal salts of sulfonated azo dyes showed that it was those salts with the least electropositive *s*-block metals that formed solvent-separated ion-pairs when crystallized from water (Kennedy *et al.*, 2009).

An exception to the general rule is the disulfonated naphthalene derived dye Orange G, which is found to give robust crystals. Thus a number of crystal structures of its metal salts were determined (Ojala *et al.*, 1994; Kennedy *et al.*, 2006). Structures with no metal-sulfonate bonds were found for the Mg (two phases), Ca and Li salts of Orange G (Ojala *et al.*, 1994; Kennedy *et al.*, 2006). Here we describe a transition metal salt of Orange G, *viz.* the Co(II) salt hexaaqua-cobalt(II) 7-oxo-8-(phenylhydrazono)-7,8-dihydronaphthalene-1,3-disulfonate tetrahydrate, [Co(OH_2_)_6_][C_16_H_10_N_2_O_7_S_2_]^.^4H_2_O. This conforms to literature expectations and forms the expected solvent-separated ion-pair type structure. Indeed, its structure is closely related to that of the Mg phase with 3.33 lattice water molecules reported by Kennedy *et al.* (2006).

The asymmetric unit contains three different molecular species with *Z*' = 3, though both the octahedral [Co(OH_2_)_6_] cations and the azo anions are found to have similar configurations throughout. The anions are found to exist as the hydrazone tautomer. The internal hydrogen leading to a 6-membered ring (see O7-C2-C1-N1-N2-H in Fig. 1) favours approximate planarity, but the steric bulk of the 1-sulfonate competes against this. Overall, the azo N-atoms lie in plane with the phenyl ring (maximum displacement from plane 0.058 (7) Å) and somewhat out of the plane of the naphthalene group (maximum displacement from plane 0.454 (6) Å). There is a small angle between the least squares planes of the two ring systems (range 14.13 (9) to 15.27 (8) °).

Expanding the contents of the asymmetric unit (Fig. 2) gives a layered packing motif with hydrophilic ([Co(H_2_O)]_6_, water, —SO_3_ and ketone O moieties) and hydrophobic layers alternating along the *b* direction (Fig. 3). Within the hydrophilic layer, all thirty independent water molecules use both H atoms as hydrogen bond donors. All the azo anion's O atoms accept hydrogen bonds as do all the free water molecules. Unusually, two of the water molecules bound to Co (O13W and O10W) also act as hydrogen bond acceptors. In the hydrophobic layer, all the aromatic rings lie parallel, with π···π contacts connecting them along the *a* direction with a closest interplanar interaction of 3.371 (5) Å.

### Experimental

Addition of a slight excess of Co(NO_3_)_2_ (0.167 g, 0.571 mmol), in aqueous solution, to a near saturated aqueous solution of the sodium salt of Orange G (0.188 g, 0.405 mmol) gave an orange precipitate. This was isolated by filtration and recrystallized from hot water to yield large crystals of the Co(II) salt of Orange G (0.188 g, 84% yield). λmax: 785 nm. IR[KBr]: 3461, 1623, 1497, 1423, 1283, 1215, 1142, 1027, 987, 762, 640, 508 cm^-1^. Raman (solid, 633 nm excitation): 1594, 1494, 1427, 1377, 1329, 1305, 1240, 1178 cm^-1^.

### Refinement

The position of the nitrogen-bound H atoms were refined freely, but the positions of the water H atoms were restrained such that O—H and H···H distances approximated 0.88 and 1.33 Å, respectively. The aromatic H atoms were placed in calculated positions and refined in riding modes with C—H = 0.95 Å. All *U*_iso_(H) values were set to 1.2*U*_eq_ of their parent non-H atom. Ten low angle reflections were omitted from the final refinement, all had extremely low observed F^2^ values as compared to calculated F^2^.

### Crystal data

**Table:**

[Co(H_2_O)_6_](C_16_H_10_N_2_O_7_S_2_)·4H_2_O	*Z* = 6
*M**_r_* = 645.47	*F*(000) = 2010
Triclinic, *P*1	*D*_x_ = 1.687 Mg m^−^^3^
Hall symbol: -P 1	Mo *K*α radiation, λ = 0.71073 Å
*a* = 10.0768 (3) Å	Cell parameters from 207115 reflections
*b* = 14.6463 (5) Å	θ = 1.0–27.1°
*c* = 26.3881 (8) Å	µ = 0.92 mm^−^^1^
α = 93.470 (2)°	*T* = 123 K
β = 90.813 (1)°	Fragment cut from large block, orange-red
γ = 101.175 (1)°	0.35 × 0.22 × 0.06 mm
*V* = 3812.4 (2) Å^3^	

### Data collection

**Table:**

Nonius KappaCCD diffractometer	16529 independent reflections
Radiation source: fine-focus sealed tube	10980 reflections with *I* > 2σ(*I*)
graphite	*R*_int_ = 0.086
ω and φ scans	θ_max_ = 27.0°, θ_min_ = 3.0°
Absorption correction: multi-scan (*SADABS*; Sheldrick, 2000)	*h* = −12→12
*T*_min_ = 0.683, *T*_max_ = 1.000	*k* = −18→18
63505 measured reflections	*l* = −33→33

### Refinement

**Table:**

Refinement on *F*^2^	Primary atom site location: structure-invariant direct methods
Least-squares matrix: full	Secondary atom site location: difference Fourier map
*R*[*F*^2^ > 2σ(*F*^2^)] = 0.056	Hydrogen site location: inferred from neighbouring sites
*wR*(*F*^2^) = 0.150	H atoms treated by a mixture of independent and constrained refinement
*S* = 1.03	*w* = 1/[σ^2^(*F*_o_^2^) + (0.0709*P*)^2^ + 4.7352*P*] where *P* = (*F*_o_^2^ + 2*F*_c_^2^)/3
16529 reflections	(Δ/σ)_max_ = 0.001
1216 parameters	Δρ_max_ = 0.95 e Å^−^^3^
90 restraints	Δρ_min_ = −1.02 e Å^−^^3^

### Special details

**Table:**

Geometry. All e.s.d.'s (except the e.s.d. in the dihedral angle between two l.s. planes) are estimated using the full covariance matrix. The cell e.s.d.'s are taken into account individually in the estimation of e.s.d.'s in distances, angles and torsion angles; correlations between e.s.d.'s in cell parameters are only used when they are defined by crystal symmetry. An approximate (isotropic) treatment of cell e.s.d.'s is used for estimating e.s.d.'s involving l.s. planes.
Refinement. Refinement of *F*^2^ against ALL reflections. The weighted *R*-factor *wR* and goodness of fit *S* are based on *F*^2^, conventional *R*-factors *R* are based on *F*, with *F* set to zero for negative *F*^2^. The threshold expression of *F*^2^ > σ(*F*^2^) is used only for calculating *R*-factors(gt) *etc*. and is not relevant to the choice of reflections for refinement. *R*-factors based on *F*^2^ are statistically about twice as large as those based on *F*, and *R*- factors based on ALL data will be even larger.

### Fractional atomic coordinates and isotropic or equivalent isotropic displacement parameters (Å^2^)

**Table:**

	*x*	*y*	*z*	*U*_iso_*/*U*_eq_	
Co1	0.55837 (5)	0.50041 (3)	0.767621 (19)	0.01612 (13)	
Co2	1.09697 (5)	0.47172 (4)	0.889819 (19)	0.01716 (13)	
Co3	0.76533 (5)	0.49375 (3)	0.574876 (19)	0.01670 (13)	
S1	0.96481 (9)	0.73184 (6)	0.78316 (3)	0.0152 (2)	
S2	0.77827 (10)	0.70362 (7)	0.97236 (3)	0.0185 (2)	
S3	0.68205 (9)	0.24597 (6)	0.88288 (3)	0.0153 (2)	
S4	0.88476 (9)	0.25429 (6)	0.69503 (3)	0.0172 (2)	
S5	0.63595 (9)	0.74418 (6)	0.44610 (3)	0.0162 (2)	
S6	0.46525 (10)	0.72989 (6)	0.63783 (4)	0.0186 (2)	
O1	0.9712 (3)	0.63918 (17)	0.80008 (9)	0.0186 (6)	
O2	0.8563 (3)	0.72713 (18)	0.74589 (9)	0.0195 (6)	
O1W	0.4973 (3)	0.37083 (18)	0.79310 (11)	0.0232 (6)	
H1W	0.415 (2)	0.342 (3)	0.7953 (15)	0.028*	
H2W	0.540 (3)	0.350 (3)	0.8171 (12)	0.028*	
O3	1.0961 (3)	0.77837 (17)	0.76568 (10)	0.0198 (6)	
O2W	0.5992 (3)	0.63442 (19)	0.74398 (10)	0.0225 (6)	
H3W	0.680 (2)	0.668 (3)	0.7485 (13)	0.027*	
H4W	0.585 (4)	0.639 (3)	0.7117 (8)	0.027*	
O4	0.8326 (3)	0.61801 (18)	0.96543 (10)	0.0249 (6)	
O3W	0.7601 (3)	0.48354 (19)	0.76745 (11)	0.0247 (6)	
H5W	0.828 (3)	0.527 (2)	0.7756 (15)	0.030*	
H6W	0.792 (4)	0.446 (2)	0.7475 (13)	0.030*	
O5	0.6328 (3)	0.68611 (19)	0.96374 (10)	0.0257 (6)	
O4W	0.6049 (3)	0.56462 (19)	0.84107 (10)	0.0212 (6)	
H7W	0.552 (4)	0.604 (2)	0.8462 (14)	0.025*	
H8W	0.593 (4)	0.532 (2)	0.8668 (11)	0.025*	
O6	0.8210 (3)	0.75364 (19)	1.02103 (9)	0.0228 (6)	
O5W	0.3525 (3)	0.51391 (19)	0.77254 (10)	0.0225 (6)	
H9W	0.338 (4)	0.5708 (16)	0.7693 (14)	0.027*	
H10W	0.314 (4)	0.503 (2)	0.8009 (10)	0.027*	
O7	1.0901 (3)	1.11905 (18)	0.79352 (10)	0.0228 (6)	
O6W	0.5178 (3)	0.4498 (2)	0.69298 (10)	0.0257 (6)	
H11W	0.440 (2)	0.422 (3)	0.6801 (14)	0.031*	
H12W	0.572 (3)	0.432 (3)	0.6706 (13)	0.031*	
O8	0.7820 (3)	0.25815 (17)	0.92442 (9)	0.0192 (6)	
O7W	1.0419 (3)	0.33513 (19)	0.91018 (11)	0.0227 (6)	
H13W	1.083 (3)	0.308 (3)	0.9312 (13)	0.027*	
H14W	0.9581 (19)	0.310 (3)	0.9125 (15)	0.027*	
O9	0.5494 (2)	0.19767 (18)	0.89640 (10)	0.0205 (6)	
O8W	1.1558 (3)	0.60918 (19)	0.87249 (10)	0.0227 (6)	
H15W	1.111 (3)	0.623 (3)	0.8474 (12)	0.027*	
H16W	1.237 (2)	0.639 (3)	0.8684 (15)	0.027*	
O10	0.6780 (3)	0.33652 (17)	0.86250 (9)	0.0190 (6)	
O9W	1.1383 (3)	0.5181 (2)	0.96528 (10)	0.0250 (6)	
H17W	1.186 (4)	0.5688 (17)	0.9799 (14)	0.030*	
H18W	1.132 (4)	0.480 (2)	0.9885 (12)	0.030*	
O11	0.8454 (3)	0.34519 (18)	0.70179 (10)	0.0246 (6)	
O10W	1.2957 (3)	0.44753 (19)	0.87963 (10)	0.0225 (6)	
H19W	1.360 (3)	0.473 (2)	0.9008 (13)	0.027*	
H20W	1.304 (4)	0.3896 (13)	0.8779 (15)	0.027*	
O12	0.8292 (3)	0.20489 (19)	0.64750 (9)	0.0229 (6)	
O11W	1.0572 (3)	0.42212 (19)	0.81367 (10)	0.0223 (6)	
H21W	1.105 (4)	0.380 (2)	0.8056 (14)	0.027*	
H22W	1.071 (4)	0.459 (2)	0.7898 (12)	0.027*	
O13	1.0300 (3)	0.26048 (19)	0.70050 (10)	0.0238 (6)	
O12W	0.8971 (3)	0.49024 (19)	0.89176 (11)	0.0223 (6)	
H23W	0.863 (4)	0.528 (2)	0.9108 (13)	0.027*	
H24W	0.828 (3)	0.447 (2)	0.8840 (15)	0.027*	
O14	0.5746 (3)	−0.13949 (17)	0.88684 (10)	0.0208 (6)	
O13W	0.6923 (3)	0.38392 (18)	0.61962 (10)	0.0227 (6)	
H25W	0.750 (3)	0.359 (2)	0.6361 (14)	0.027*	
H26W	0.634 (3)	0.3351 (19)	0.6094 (15)	0.027*	
O15	0.7666 (3)	0.78774 (17)	0.42708 (10)	0.0214 (6)	
O14W	0.8255 (3)	0.6108 (2)	0.53505 (11)	0.0265 (7)	
H27W	0.780 (3)	0.627 (3)	0.5104 (12)	0.032*	
H28W	0.907 (2)	0.641 (3)	0.5306 (15)	0.032*	
O16	0.5229 (3)	0.74048 (18)	0.41075 (9)	0.0205 (6)	
O15W	0.7972 (3)	0.58080 (19)	0.64190 (11)	0.0253 (6)	
H29W	0.748 (3)	0.624 (2)	0.6445 (16)	0.030*	
H30W	0.874 (2)	0.610 (2)	0.6552 (16)	0.030*	
O17	0.6399 (3)	0.65155 (17)	0.46390 (10)	0.0203 (6)	
O16W	0.5631 (3)	0.51140 (19)	0.56506 (10)	0.0202 (6)	
H31W	0.529 (3)	0.547 (2)	0.5869 (12)	0.024*	
H32W	0.493 (3)	0.4661 (19)	0.5590 (14)	0.024*	
O18	0.5391 (3)	0.65342 (19)	0.63945 (10)	0.0257 (6)	
O17W	0.7270 (3)	0.4100 (2)	0.50873 (10)	0.0242 (6)	
H33W	0.784 (3)	0.375 (2)	0.5021 (14)	0.029*	
H34W	0.729 (4)	0.441 (2)	0.4827 (11)	0.029*	
O19	0.3240 (3)	0.6965 (2)	0.62217 (11)	0.0280 (7)	
O18W	0.9590 (3)	0.46353 (19)	0.58642 (12)	0.0267 (7)	
H35W	0.970 (4)	0.4055 (13)	0.5839 (17)	0.032*	
H36W	1.040 (2)	0.493 (2)	0.5890 (17)	0.032*	
O20	0.4800 (3)	0.78783 (18)	0.68518 (10)	0.0253 (6)	
O19W	0.4276 (3)	0.68555 (18)	0.85871 (10)	0.0223 (6)	
H37W	0.478 (4)	0.7386 (18)	0.8689 (13)	0.027*	
H38W	0.403 (4)	0.698 (2)	0.8286 (9)	0.027*	
O21	0.7537 (3)	1.13329 (17)	0.44848 (9)	0.0203 (6)	
O20W	1.0664 (3)	0.6635 (2)	0.66736 (11)	0.0264 (7)	
H39W	1.072 (4)	0.7158 (19)	0.6845 (13)	0.032*	
H40W	1.137 (3)	0.678 (3)	0.6484 (13)	0.032*	
O21W	0.3253 (3)	0.7000 (2)	0.76328 (10)	0.0238 (6)	
H41W	0.377 (3)	0.732 (3)	0.7419 (13)	0.029*	
H42W	0.253 (2)	0.723 (3)	0.7602 (15)	0.029*	
O22W	1.0745 (3)	0.4915 (2)	0.71520 (11)	0.0283 (7)	
H43W	0.996 (3)	0.460 (2)	0.7045 (16)	0.034*	
H44W	1.072 (4)	0.5461 (15)	0.7041 (16)	0.034*	
O23W	0.5375 (3)	0.4923 (2)	0.93519 (10)	0.0265 (7)	
H45W	0.580 (4)	0.5470 (14)	0.9478 (15)	0.032*	
H46W	0.579 (4)	0.456 (2)	0.9506 (15)	0.032*	
O24W	0.6319 (3)	0.35913 (19)	0.99677 (11)	0.0273 (7)	
H47W	0.557 (3)	0.329 (2)	1.0089 (15)	0.033*	
H48W	0.662 (4)	0.311 (2)	0.9830 (15)	0.033*	
O25W	0.3178 (3)	0.2676 (2)	0.88730 (11)	0.0263 (7)	
H49W	0.281 (3)	0.252 (3)	0.9161 (10)	0.032*	
H50W	0.398 (2)	0.253 (3)	0.8923 (15)	0.032*	
O26W	0.2200 (3)	0.29879 (18)	0.78462 (11)	0.0249 (6)	
H51W	0.178 (4)	0.283 (3)	0.7550 (9)	0.030*	
H52W	0.192 (4)	0.2477 (19)	0.7998 (12)	0.030*	
O27W	0.2554 (3)	0.3950 (2)	0.66159 (12)	0.0332 (7)	
H53W	0.231 (4)	0.3340 (13)	0.6620 (17)	0.040*	
H54W	0.201 (4)	0.414 (3)	0.6828 (14)	0.040*	
O28W	0.9989 (3)	0.27951 (19)	0.57343 (10)	0.0231 (6)	
H55W	0.949 (3)	0.238 (2)	0.5906 (14)	0.028*	
H56W	1.076 (2)	0.265 (3)	0.5779 (15)	0.028*	
O29W	0.2375 (3)	0.5172 (2)	0.58405 (11)	0.0281 (7)	
H57W	0.276 (4)	0.5714 (15)	0.5985 (15)	0.034*	
H58W	0.263 (4)	0.479 (2)	0.6045 (14)	0.034*	
O30W	0.9102 (3)	0.30277 (19)	0.47948 (11)	0.0237 (6)	
H59W	0.941 (4)	0.286 (2)	0.5067 (10)	0.028*	
H60W	0.853 (3)	0.252 (2)	0.4683 (13)	0.028*	
N1	0.9773 (3)	0.9315 (2)	0.76101 (11)	0.0156 (7)	
N2	1.0132 (3)	0.9896 (2)	0.72600 (12)	0.0179 (7)	
H1N	1.049 (4)	1.047 (3)	0.7371 (15)	0.021*	
N3	0.6850 (3)	0.0514 (2)	0.91295 (11)	0.0162 (7)	
N4	0.6534 (3)	−0.0023 (2)	0.95014 (12)	0.0171 (7)	
H2N	0.615 (4)	−0.052 (3)	0.9427 (16)	0.021*	
N5	0.6475 (3)	0.9429 (2)	0.42117 (11)	0.0159 (7)	
N6	0.6777 (3)	0.9984 (2)	0.38471 (12)	0.0175 (7)	
H3N	0.713 (4)	1.051 (3)	0.3951 (16)	0.021*	
C1	0.9985 (4)	0.9627 (3)	0.81018 (14)	0.0169 (8)	
C2	1.0459 (4)	1.0606 (3)	0.82555 (15)	0.0209 (8)	
C3	1.0344 (4)	1.0913 (3)	0.87812 (14)	0.0203 (8)	
H3	1.0637	1.1552	0.8888	0.024*	
C4	0.9824 (4)	1.0297 (3)	0.91211 (14)	0.0194 (8)	
H4	0.9724	1.0522	0.9461	0.023*	
C5	0.9413 (4)	0.9311 (3)	0.89878 (14)	0.0179 (8)	
C6	0.9548 (3)	0.8952 (3)	0.84803 (13)	0.0150 (8)	
C7	0.9268 (3)	0.7966 (2)	0.83949 (13)	0.0147 (8)	
C8	0.8724 (4)	0.7397 (3)	0.87726 (14)	0.0164 (8)	
H8	0.8492	0.6741	0.8702	0.020*	
C9	0.8516 (4)	0.7778 (3)	0.92547 (13)	0.0168 (8)	
C10	0.8892 (4)	0.8724 (3)	0.93646 (14)	0.0186 (8)	
H10	0.8796	0.8979	0.9699	0.022*	
C11	0.9855 (4)	0.9627 (3)	0.67409 (14)	0.0185 (8)	
C12	1.0236 (4)	1.0308 (3)	0.63942 (15)	0.0207 (8)	
H12	1.0644	1.0927	0.6510	0.025*	
C13	1.0014 (4)	1.0071 (3)	0.58792 (15)	0.0247 (9)	
H13	1.0264	1.0533	0.5642	0.030*	
C14	0.9431 (4)	0.9169 (3)	0.57058 (15)	0.0258 (9)	
H14	0.9294	0.9011	0.5352	0.031*	
C15	0.9049 (4)	0.8498 (3)	0.60534 (15)	0.0257 (9)	
H15	0.8647	0.7879	0.5935	0.031*	
C16	0.9246 (4)	0.8720 (3)	0.65739 (15)	0.0235 (9)	
H16	0.8971	0.8261	0.6810	0.028*	
C17	0.6620 (4)	0.0152 (3)	0.86506 (14)	0.0158 (8)	
C18	0.6150 (4)	−0.0844 (3)	0.85263 (14)	0.0184 (8)	
C19	0.6216 (4)	−0.1191 (3)	0.80070 (14)	0.0187 (8)	
H19	0.5901	−0.1836	0.7917	0.022*	
C20	0.6717 (4)	−0.0616 (3)	0.76451 (14)	0.0185 (8)	
H20	0.6789	−0.0873	0.7310	0.022*	
C21	0.7145 (4)	0.0373 (2)	0.77505 (14)	0.0156 (8)	
C22	0.7032 (4)	0.0783 (3)	0.82477 (13)	0.0152 (8)	
C23	0.7332 (3)	0.1779 (3)	0.83067 (13)	0.0145 (8)	
C24	0.7888 (4)	0.2306 (3)	0.79142 (14)	0.0174 (8)	
H24	0.8140	0.2964	0.7968	0.021*	
C25	0.8081 (4)	0.1874 (3)	0.74396 (13)	0.0160 (8)	
C26	0.7663 (4)	0.0926 (3)	0.73539 (14)	0.0176 (8)	
H26	0.7725	0.0642	0.7024	0.021*	
C27	0.6836 (4)	0.0304 (3)	1.00097 (14)	0.0181 (8)	
C28	0.6471 (4)	−0.0325 (3)	1.03830 (14)	0.0217 (9)	
H28	0.6060	−0.0955	1.0293	0.026*	
C29	0.6721 (4)	−0.0012 (3)	1.08900 (15)	0.0260 (9)	
H29	0.6483	−0.0435	1.1148	0.031*	
C30	0.7311 (4)	0.0907 (3)	1.10243 (15)	0.0242 (9)	
H30	0.7458	0.1117	1.1372	0.029*	
C31	0.7686 (4)	0.1519 (3)	1.06474 (14)	0.0231 (9)	
H31	0.8102	0.2148	1.0738	0.028*	
C32	0.7460 (4)	0.1223 (3)	1.01391 (15)	0.0235 (9)	
H32	0.7729	0.1642	0.9882	0.028*	
C33	0.6712 (4)	0.9774 (2)	0.46917 (14)	0.0159 (8)	
C34	0.7143 (4)	1.0771 (3)	0.48250 (14)	0.0177 (8)	
C35	0.7050 (4)	1.1104 (3)	0.53434 (14)	0.0174 (8)	
H35	0.7307	1.1752	0.5436	0.021*	
C36	0.6603 (4)	1.0508 (3)	0.57003 (15)	0.0194 (8)	
H36	0.6534	1.0752	0.6039	0.023*	
C37	0.6226 (4)	0.9514 (2)	0.55889 (14)	0.0160 (8)	
C38	0.6340 (4)	0.9126 (2)	0.50909 (13)	0.0149 (8)	
C39	0.6059 (3)	0.8128 (2)	0.50205 (13)	0.0145 (7)	
C40	0.5558 (4)	0.7592 (3)	0.54198 (14)	0.0166 (8)	
H40	0.5336	0.6932	0.5367	0.020*	
C41	0.5377 (4)	0.8009 (3)	0.58955 (14)	0.0168 (8)	
C42	0.5753 (4)	0.8957 (3)	0.59870 (14)	0.0165 (8)	
H42	0.5692	0.9234	0.6319	0.020*	
C43	0.6465 (4)	0.9681 (3)	0.33345 (14)	0.0186 (8)	
C44	0.6822 (4)	1.0328 (3)	0.29734 (14)	0.0203 (8)	
H44	0.7229	1.0955	0.3074	0.024*	
C45	0.6579 (4)	1.0052 (3)	0.24633 (15)	0.0266 (9)	
H45	0.6823	1.0491	0.2214	0.032*	
C46	0.5985 (4)	0.9142 (3)	0.23155 (15)	0.0286 (10)	
H46	0.5824	0.8956	0.1965	0.034*	
C47	0.5624 (4)	0.8500 (3)	0.26805 (15)	0.0251 (9)	
H47	0.5213	0.7875	0.2578	0.030*	
C48	0.5859 (4)	0.8763 (3)	0.31951 (15)	0.0222 (9)	
H48	0.5611	0.8325	0.3445	0.027*	

### Atomic displacement parameters (Å^2^)

**Table:**

	*U*^11^	*U*^22^	*U*^33^	*U*^12^	*U*^13^	*U*^23^
Co1	0.0150 (3)	0.0196 (3)	0.0138 (3)	0.0030 (2)	0.0032 (2)	0.0019 (2)
Co2	0.0152 (3)	0.0232 (3)	0.0135 (3)	0.0035 (2)	0.0034 (2)	0.0043 (2)
Co3	0.0153 (3)	0.0196 (3)	0.0153 (3)	0.0029 (2)	0.0037 (2)	0.0028 (2)
S1	0.0163 (5)	0.0174 (5)	0.0120 (5)	0.0032 (4)	0.0039 (4)	0.0005 (4)
S2	0.0193 (5)	0.0241 (5)	0.0123 (5)	0.0043 (4)	0.0045 (4)	0.0013 (4)
S3	0.0158 (5)	0.0178 (5)	0.0127 (5)	0.0036 (4)	0.0040 (4)	0.0016 (4)
S4	0.0177 (5)	0.0209 (5)	0.0129 (5)	0.0024 (4)	0.0048 (4)	0.0037 (4)
S5	0.0175 (5)	0.0163 (5)	0.0143 (5)	0.0019 (4)	0.0027 (4)	0.0014 (4)
S6	0.0223 (5)	0.0190 (5)	0.0143 (5)	0.0021 (4)	0.0040 (4)	0.0040 (4)
O1	0.0192 (14)	0.0207 (14)	0.0161 (14)	0.0041 (11)	0.0057 (11)	0.0006 (11)
O2	0.0209 (14)	0.0225 (14)	0.0132 (13)	−0.0002 (11)	−0.0001 (11)	0.0006 (11)
O1W	0.0194 (15)	0.0216 (15)	0.0287 (17)	0.0028 (12)	0.0003 (12)	0.0073 (12)
O3	0.0170 (14)	0.0229 (14)	0.0202 (14)	0.0053 (11)	0.0076 (11)	0.0018 (11)
O2W	0.0181 (15)	0.0300 (16)	0.0196 (15)	0.0036 (12)	0.0027 (12)	0.0063 (12)
O4	0.0376 (17)	0.0276 (15)	0.0126 (14)	0.0118 (13)	0.0103 (12)	0.0059 (11)
O3W	0.0162 (15)	0.0243 (16)	0.0325 (17)	0.0035 (12)	0.0029 (13)	−0.0072 (13)
O5	0.0185 (15)	0.0357 (17)	0.0226 (15)	0.0030 (12)	0.0063 (12)	0.0061 (13)
O4W	0.0228 (15)	0.0264 (16)	0.0149 (14)	0.0058 (12)	0.0034 (12)	0.0029 (12)
O6	0.0253 (15)	0.0307 (16)	0.0118 (14)	0.0043 (12)	0.0041 (11)	0.0004 (11)
O5W	0.0183 (15)	0.0272 (16)	0.0228 (16)	0.0063 (12)	0.0065 (12)	0.0023 (12)
O7	0.0242 (15)	0.0229 (15)	0.0203 (15)	0.0016 (12)	0.0037 (12)	0.0025 (12)
O6W	0.0165 (15)	0.0420 (18)	0.0171 (15)	0.0035 (13)	0.0040 (12)	−0.0041 (13)
O8	0.0224 (15)	0.0216 (14)	0.0121 (13)	0.0009 (11)	0.0002 (11)	0.0014 (11)
O7W	0.0181 (14)	0.0251 (15)	0.0257 (16)	0.0040 (12)	−0.0005 (12)	0.0090 (12)
O9	0.0144 (14)	0.0244 (15)	0.0227 (15)	0.0028 (11)	0.0078 (11)	0.0028 (11)
O8W	0.0183 (15)	0.0273 (16)	0.0225 (16)	0.0030 (12)	−0.0003 (12)	0.0069 (12)
O10	0.0225 (15)	0.0184 (14)	0.0171 (14)	0.0063 (11)	0.0033 (11)	0.0017 (11)
O9W	0.0329 (17)	0.0269 (16)	0.0133 (14)	0.0004 (13)	0.0009 (12)	0.0032 (12)
O11	0.0300 (16)	0.0240 (15)	0.0211 (15)	0.0067 (12)	0.0093 (12)	0.0053 (12)
O10W	0.0179 (15)	0.0255 (15)	0.0240 (16)	0.0038 (12)	0.0009 (12)	0.0011 (12)
O12	0.0224 (15)	0.0334 (16)	0.0108 (14)	0.0002 (12)	0.0021 (11)	0.0006 (11)
O11W	0.0244 (16)	0.0281 (16)	0.0166 (15)	0.0090 (12)	0.0036 (12)	0.0045 (12)
O13	0.0164 (14)	0.0359 (17)	0.0181 (15)	0.0012 (12)	0.0052 (11)	0.0065 (12)
O12W	0.0160 (14)	0.0254 (16)	0.0255 (16)	0.0054 (11)	0.0037 (12)	−0.0026 (12)
O14	0.0243 (15)	0.0205 (14)	0.0170 (14)	0.0017 (11)	0.0024 (11)	0.0052 (11)
O13W	0.0208 (15)	0.0256 (15)	0.0207 (15)	0.0009 (12)	−0.0010 (12)	0.0058 (12)
O15	0.0203 (14)	0.0211 (14)	0.0243 (15)	0.0064 (11)	0.0083 (12)	0.0046 (12)
O14W	0.0197 (15)	0.0325 (17)	0.0268 (17)	0.0001 (13)	0.0014 (13)	0.0146 (13)
O16	0.0230 (15)	0.0219 (14)	0.0146 (14)	−0.0001 (11)	−0.0019 (11)	0.0006 (11)
O15W	0.0241 (16)	0.0283 (16)	0.0222 (16)	0.0038 (13)	0.0019 (13)	−0.0054 (13)
O17	0.0244 (15)	0.0169 (14)	0.0197 (14)	0.0044 (11)	0.0045 (12)	0.0003 (11)
O16W	0.0155 (14)	0.0225 (15)	0.0234 (15)	0.0054 (11)	0.0037 (12)	0.0009 (12)
O18	0.0366 (17)	0.0268 (15)	0.0159 (14)	0.0102 (13)	0.0074 (12)	0.0030 (12)
O17W	0.0265 (16)	0.0307 (17)	0.0164 (15)	0.0079 (13)	0.0040 (12)	0.0015 (12)
O19	0.0237 (16)	0.0337 (17)	0.0247 (16)	0.0000 (13)	0.0079 (12)	0.0041 (13)
O18W	0.0138 (14)	0.0236 (15)	0.0425 (19)	0.0035 (12)	0.0009 (13)	0.0005 (14)
O20	0.0398 (18)	0.0207 (14)	0.0157 (14)	0.0057 (13)	0.0097 (13)	0.0008 (11)
O19W	0.0248 (16)	0.0216 (15)	0.0190 (15)	0.0005 (12)	0.0036 (12)	0.0010 (12)
O21	0.0252 (15)	0.0195 (14)	0.0151 (14)	0.0005 (11)	0.0027 (11)	0.0049 (11)
O20W	0.0236 (16)	0.0305 (17)	0.0235 (16)	0.0020 (13)	0.0092 (12)	−0.0023 (13)
O21W	0.0200 (15)	0.0330 (17)	0.0221 (15)	0.0115 (13)	0.0082 (12)	0.0091 (12)
O22W	0.0252 (16)	0.0326 (17)	0.0262 (17)	0.0024 (13)	−0.0001 (13)	0.0060 (13)
O23W	0.0321 (17)	0.0263 (16)	0.0204 (16)	0.0046 (13)	−0.0044 (13)	0.0010 (12)
O24W	0.0295 (17)	0.0306 (17)	0.0218 (16)	0.0068 (13)	0.0076 (13)	−0.0029 (13)
O25W	0.0221 (16)	0.0370 (17)	0.0234 (16)	0.0113 (13)	0.0080 (13)	0.0097 (13)
O26W	0.0268 (16)	0.0221 (15)	0.0246 (16)	0.0008 (12)	0.0029 (13)	0.0039 (12)
O27W	0.0253 (17)	0.0468 (19)	0.0255 (17)	0.0017 (15)	0.0047 (13)	0.0035 (15)
O28W	0.0204 (15)	0.0300 (16)	0.0203 (15)	0.0060 (12)	0.0057 (12)	0.0075 (12)
O29W	0.0259 (17)	0.0304 (17)	0.0262 (17)	0.0008 (13)	0.0032 (13)	0.0009 (13)
O30W	0.0247 (16)	0.0246 (15)	0.0210 (16)	0.0027 (12)	0.0042 (12)	0.0006 (12)
N1	0.0140 (16)	0.0193 (16)	0.0146 (16)	0.0052 (13)	0.0041 (13)	0.0029 (13)
N2	0.0183 (17)	0.0189 (17)	0.0162 (17)	0.0022 (14)	0.0059 (13)	0.0029 (14)
N3	0.0139 (16)	0.0202 (16)	0.0163 (17)	0.0064 (13)	0.0044 (13)	0.0033 (13)
N4	0.0190 (18)	0.0183 (17)	0.0134 (17)	0.0013 (14)	0.0029 (13)	0.0036 (14)
N5	0.0136 (16)	0.0207 (17)	0.0150 (17)	0.0063 (13)	0.0023 (13)	0.0034 (13)
N6	0.0183 (17)	0.0203 (17)	0.0135 (17)	0.0012 (14)	0.0032 (13)	0.0047 (14)
C1	0.0147 (19)	0.0196 (19)	0.017 (2)	0.0034 (15)	0.0024 (15)	0.0032 (15)
C2	0.015 (2)	0.024 (2)	0.023 (2)	0.0029 (16)	0.0000 (16)	0.0051 (17)
C3	0.017 (2)	0.022 (2)	0.021 (2)	0.0041 (16)	−0.0003 (16)	−0.0024 (16)
C4	0.017 (2)	0.025 (2)	0.017 (2)	0.0050 (16)	0.0024 (16)	−0.0014 (16)
C5	0.0112 (18)	0.024 (2)	0.018 (2)	0.0034 (15)	0.0020 (15)	−0.0004 (16)
C6	0.0089 (18)	0.023 (2)	0.0134 (19)	0.0035 (15)	0.0026 (14)	0.0017 (15)
C7	0.0118 (18)	0.0209 (19)	0.0113 (18)	0.0025 (15)	0.0014 (14)	0.0012 (15)
C8	0.0151 (19)	0.0169 (19)	0.017 (2)	0.0026 (15)	0.0009 (15)	0.0002 (15)
C9	0.0157 (19)	0.027 (2)	0.0097 (18)	0.0068 (16)	0.0034 (15)	0.0025 (15)
C10	0.019 (2)	0.023 (2)	0.0145 (19)	0.0048 (16)	0.0039 (15)	−0.0002 (16)
C11	0.0147 (19)	0.026 (2)	0.016 (2)	0.0073 (16)	0.0050 (15)	0.0046 (16)
C12	0.018 (2)	0.023 (2)	0.022 (2)	0.0050 (16)	0.0054 (16)	0.0047 (16)
C13	0.022 (2)	0.035 (2)	0.020 (2)	0.0091 (18)	0.0056 (17)	0.0130 (18)
C14	0.023 (2)	0.040 (3)	0.018 (2)	0.0135 (19)	0.0045 (17)	0.0043 (18)
C15	0.025 (2)	0.030 (2)	0.022 (2)	0.0063 (18)	0.0020 (18)	0.0004 (18)
C16	0.026 (2)	0.026 (2)	0.020 (2)	0.0054 (17)	0.0068 (17)	0.0067 (17)
C17	0.0149 (19)	0.020 (2)	0.0131 (19)	0.0050 (15)	0.0040 (15)	0.0018 (15)
C18	0.0142 (19)	0.022 (2)	0.020 (2)	0.0050 (15)	0.0016 (15)	0.0026 (16)
C19	0.019 (2)	0.019 (2)	0.016 (2)	0.0010 (16)	0.0005 (16)	−0.0032 (16)
C20	0.0160 (19)	0.023 (2)	0.016 (2)	0.0036 (16)	0.0032 (15)	−0.0013 (16)
C21	0.0143 (19)	0.0183 (19)	0.0148 (19)	0.0041 (15)	0.0042 (15)	0.0024 (15)
C22	0.0116 (18)	0.022 (2)	0.0124 (18)	0.0032 (15)	0.0023 (14)	0.0035 (15)
C23	0.0113 (18)	0.023 (2)	0.0091 (18)	0.0041 (15)	0.0007 (14)	−0.0003 (15)
C24	0.0147 (19)	0.020 (2)	0.018 (2)	0.0030 (15)	0.0012 (15)	0.0032 (15)
C25	0.0143 (19)	0.024 (2)	0.0101 (18)	0.0044 (15)	0.0048 (14)	0.0047 (15)
C26	0.017 (2)	0.025 (2)	0.0108 (19)	0.0048 (16)	0.0011 (15)	0.0024 (15)
C27	0.0153 (19)	0.027 (2)	0.0134 (19)	0.0061 (16)	0.0029 (15)	0.0039 (16)
C28	0.020 (2)	0.026 (2)	0.018 (2)	0.0027 (17)	0.0021 (16)	0.0087 (17)
C29	0.021 (2)	0.041 (3)	0.018 (2)	0.0066 (19)	0.0059 (17)	0.0114 (18)
C30	0.019 (2)	0.043 (3)	0.013 (2)	0.0102 (19)	0.0028 (16)	0.0004 (18)
C31	0.026 (2)	0.027 (2)	0.016 (2)	0.0050 (18)	0.0009 (17)	−0.0023 (17)
C32	0.027 (2)	0.024 (2)	0.020 (2)	0.0037 (17)	0.0064 (17)	0.0068 (17)
C33	0.0134 (19)	0.0178 (19)	0.017 (2)	0.0039 (15)	0.0027 (15)	0.0024 (15)
C34	0.0132 (19)	0.020 (2)	0.021 (2)	0.0033 (15)	0.0028 (15)	0.0064 (16)
C35	0.0165 (19)	0.0167 (19)	0.018 (2)	0.0016 (15)	0.0035 (15)	0.0010 (15)
C36	0.017 (2)	0.024 (2)	0.017 (2)	0.0025 (16)	0.0016 (16)	−0.0020 (16)
C37	0.0141 (18)	0.0196 (19)	0.0146 (19)	0.0033 (15)	0.0034 (15)	0.0027 (15)
C38	0.0137 (18)	0.0180 (19)	0.0128 (19)	0.0023 (15)	0.0021 (14)	0.0021 (15)
C39	0.0111 (18)	0.0184 (19)	0.0144 (19)	0.0038 (14)	0.0007 (14)	0.0014 (15)
C40	0.0155 (19)	0.0175 (19)	0.017 (2)	0.0025 (15)	0.0027 (15)	0.0041 (15)
C41	0.0156 (19)	0.022 (2)	0.0127 (19)	0.0033 (15)	0.0029 (15)	0.0017 (15)
C42	0.0145 (19)	0.022 (2)	0.0124 (19)	0.0037 (15)	−0.0007 (15)	−0.0015 (15)
C43	0.0155 (19)	0.027 (2)	0.015 (2)	0.0076 (16)	0.0041 (15)	0.0041 (16)
C44	0.018 (2)	0.026 (2)	0.018 (2)	0.0059 (16)	0.0045 (16)	0.0047 (16)
C45	0.020 (2)	0.041 (3)	0.021 (2)	0.0102 (19)	0.0073 (17)	0.0104 (19)
C46	0.022 (2)	0.052 (3)	0.014 (2)	0.011 (2)	0.0021 (17)	0.0021 (19)
C47	0.023 (2)	0.031 (2)	0.021 (2)	0.0051 (18)	0.0008 (17)	−0.0041 (18)
C48	0.023 (2)	0.026 (2)	0.017 (2)	0.0034 (17)	0.0011 (16)	0.0029 (17)

### Geometric parameters (Å, °)

**Table:**

Co1—O1W	2.036 (3)	O27W—H54W	0.865 (18)
Co1—O2W	2.062 (3)	O28W—H55W	0.866 (18)
Co1—O6W	2.068 (3)	O28W—H56W	0.858 (18)
Co1—O3W	2.094 (3)	O29W—H57W	0.874 (18)
Co1—O4W	2.108 (3)	O29W—H58W	0.867 (18)
Co1—O5W	2.127 (3)	O30W—H59W	0.844 (18)
Co2—O8W	2.066 (3)	O30W—H60W	0.880 (18)
Co2—O9W	2.074 (3)	N1—N2	1.302 (4)
Co2—O7W	2.075 (3)	N1—C1	1.349 (5)
Co2—O12W	2.085 (3)	N2—C11	1.411 (5)
Co2—O11W	2.101 (3)	N2—H1N	0.88 (4)
Co2—O10W	2.118 (3)	N3—N4	1.298 (4)
Co3—O17W	2.059 (3)	N3—C17	1.339 (5)
Co3—O14W	2.060 (3)	N4—C27	1.406 (5)
Co3—O13W	2.078 (3)	N4—H2N	0.77 (4)
Co3—O15W	2.100 (3)	N5—N6	1.298 (4)
Co3—O18W	2.105 (3)	N5—C33	1.336 (5)
Co3—O16W	2.117 (3)	N6—C43	1.409 (5)
S1—O2	1.449 (3)	N6—H3N	0.82 (4)
S1—O3	1.461 (3)	C1—C2	1.452 (5)
S1—O1	1.467 (3)	C1—C6	1.460 (5)
S1—C7	1.800 (4)	C2—C3	1.445 (5)
S2—O5	1.450 (3)	C3—C4	1.349 (5)
S2—O6	1.456 (3)	C3—H3	0.9500
S2—O4	1.465 (3)	C4—C5	1.444 (5)
S2—C9	1.771 (4)	C4—H4	0.9500
S3—O9	1.447 (3)	C5—C10	1.395 (5)
S3—O8	1.457 (3)	C5—C6	1.427 (5)
S3—O10	1.469 (3)	C6—C7	1.420 (5)
S3—C23	1.792 (4)	C7—C8	1.388 (5)
S4—O13	1.454 (3)	C8—C9	1.393 (5)
S4—O12	1.457 (3)	C8—H8	0.9500
S4—O11	1.463 (3)	C9—C10	1.375 (5)
S4—C25	1.764 (4)	C10—H10	0.9500
S5—O16	1.453 (3)	C11—C16	1.392 (5)
S5—O15	1.457 (3)	C11—C12	1.398 (5)
S5—O17	1.470 (3)	C12—C13	1.387 (6)
S5—C39	1.798 (4)	C12—H12	0.9500
S6—O20	1.456 (3)	C13—C14	1.384 (6)
S6—O19	1.457 (3)	C13—H13	0.9500
S6—O18	1.462 (3)	C14—C15	1.389 (6)
S6—C41	1.770 (4)	C14—H14	0.9500
O1W—H1W	0.862 (18)	C15—C16	1.395 (6)
O1W—H2W	0.863 (18)	C15—H15	0.9500
O2W—H3W	0.863 (18)	C16—H16	0.9500
O2W—H4W	0.872 (18)	C17—C18	1.460 (5)
O3W—H5W	0.853 (18)	C17—C22	1.463 (5)
O3W—H6W	0.849 (18)	C18—C19	1.440 (5)
O4W—H7W	0.858 (18)	C19—C20	1.350 (5)
O4W—H8W	0.853 (18)	C19—H19	0.9500
O5W—H9W	0.882 (18)	C20—C21	1.437 (5)
O5W—H10W	0.855 (18)	C20—H20	0.9500
O7—C2	1.263 (4)	C21—C26	1.407 (5)
O6W—H11W	0.864 (18)	C21—C22	1.425 (5)
O6W—H12W	0.868 (18)	C22—C23	1.429 (5)
O7W—H13W	0.853 (18)	C23—C24	1.389 (5)
O7W—H14W	0.859 (18)	C24—C25	1.402 (5)
O8W—H15W	0.857 (18)	C24—H24	0.9500
O8W—H16W	0.858 (18)	C25—C26	1.374 (5)
O9W—H17W	0.869 (18)	C26—H26	0.9500
O9W—H18W	0.847 (18)	C27—C32	1.390 (5)
O10W—H19W	0.860 (18)	C27—C28	1.394 (5)
O10W—H20W	0.866 (18)	C28—C29	1.391 (6)
O11W—H21W	0.866 (18)	C28—H28	0.9500
O11W—H22W	0.855 (18)	C29—C30	1.385 (6)
O12W—H23W	0.852 (18)	C29—H29	0.9500
O12W—H24W	0.856 (18)	C30—C31	1.386 (5)
O14—C18	1.266 (4)	C30—H30	0.9500
O13W—H25W	0.872 (18)	C31—C32	1.387 (5)
O13W—H26W	0.862 (18)	C31—H31	0.9500
O14W—H27W	0.856 (18)	C32—H32	0.9500
O14W—H28W	0.864 (18)	C33—C34	1.459 (5)
O15W—H29W	0.870 (18)	C33—C38	1.465 (5)
O15W—H30W	0.866 (18)	C34—C35	1.435 (5)
O16W—H31W	0.870 (18)	C35—C36	1.345 (5)
O16W—H32W	0.875 (18)	C35—H35	0.9500
O17W—H33W	0.859 (18)	C36—C37	1.443 (5)
O17W—H34W	0.849 (18)	C36—H36	0.9500
O18W—H35W	0.877 (18)	C37—C42	1.402 (5)
O18W—H36W	0.846 (18)	C37—C38	1.415 (5)
O19W—H37W	0.867 (18)	C38—C39	1.434 (5)
O19W—H38W	0.870 (18)	C39—C40	1.394 (5)
O21—C34	1.271 (4)	C40—C41	1.392 (5)
O20W—H39W	0.858 (18)	C40—H40	0.9500
O20W—H40W	0.880 (18)	C41—C42	1.371 (5)
O21W—H41W	0.872 (18)	C42—H42	0.9500
O21W—H42W	0.865 (18)	C43—C44	1.387 (5)
O22W—H43W	0.872 (18)	C43—C48	1.390 (5)
O22W—H44W	0.873 (18)	C44—C45	1.387 (6)
O23W—H45W	0.877 (18)	C44—H44	0.9500
O23W—H46W	0.856 (18)	C45—C46	1.381 (6)
O24W—H47W	0.875 (18)	C45—H45	0.9500
O24W—H48W	0.877 (18)	C46—C47	1.390 (6)
O25W—H49W	0.877 (18)	C46—H46	0.9500
O25W—H50W	0.880 (18)	C47—C48	1.393 (5)
O26W—H51W	0.879 (18)	C47—H47	0.9500
O26W—H52W	0.871 (18)	C48—H48	0.9500
O27W—H53W	0.881 (18)		
			
O1W—Co1—O2W	173.61 (11)	N3—N4—C27	121.6 (3)
O1W—Co1—O6W	91.55 (12)	N3—N4—H2N	116 (3)
O2W—Co1—O6W	89.54 (12)	C27—N4—H2N	122 (3)
O1W—Co1—O3W	91.02 (11)	N6—N5—C33	118.9 (3)
O2W—Co1—O3W	95.18 (11)	N5—N6—C43	121.7 (3)
O6W—Co1—O3W	94.49 (11)	N5—N6—H3N	113 (3)
O1W—Co1—O4W	93.86 (11)	C43—N6—H3N	126 (3)
O2W—Co1—O4W	85.06 (11)	N1—C1—C2	122.3 (3)
O6W—Co1—O4W	174.58 (11)	N1—C1—C6	116.7 (3)
O3W—Co1—O4W	85.61 (11)	C2—C1—C6	120.5 (3)
O1W—Co1—O5W	86.34 (11)	O7—C2—C3	120.2 (4)
O2W—Co1—O5W	87.40 (11)	O7—C2—C1	121.2 (4)
O6W—Co1—O5W	88.23 (11)	C3—C2—C1	118.5 (3)
O3W—Co1—O5W	176.26 (11)	C4—C3—C2	120.5 (4)
O4W—Co1—O5W	91.92 (11)	C4—C3—H3	119.8
O8W—Co2—O9W	86.98 (11)	C2—C3—H3	119.8
O8W—Co2—O7W	177.60 (12)	C3—C4—C5	122.5 (4)
O9W—Co2—O7W	90.72 (11)	C3—C4—H4	118.7
O8W—Co2—O12W	89.06 (11)	C5—C4—H4	118.7
O9W—Co2—O12W	93.82 (11)	C10—C5—C6	121.2 (3)
O7W—Co2—O12W	91.79 (11)	C10—C5—C4	118.6 (3)
O8W—Co2—O11W	93.99 (11)	C6—C5—C4	120.2 (3)
O9W—Co2—O11W	178.69 (11)	C7—C6—C5	116.5 (3)
O7W—Co2—O11W	88.29 (11)	C7—C6—C1	126.2 (3)
O12W—Co2—O11W	87.07 (11)	C5—C6—C1	117.3 (3)
O8W—Co2—O10W	91.38 (11)	C8—C7—C6	120.9 (3)
O9W—Co2—O10W	92.32 (11)	C8—C7—S1	113.0 (3)
O7W—Co2—O10W	88.01 (11)	C6—C7—S1	126.0 (3)
O12W—Co2—O10W	173.86 (11)	C7—C8—C9	120.7 (3)
O11W—Co2—O10W	86.78 (11)	C7—C8—H8	119.7
O17W—Co3—O14W	91.59 (12)	C9—C8—H8	119.7
O17W—Co3—O13W	92.58 (11)	C10—C9—C8	119.9 (3)
O14W—Co3—O13W	173.89 (12)	C10—C9—S2	120.5 (3)
O17W—Co3—O15W	177.78 (11)	C8—C9—S2	119.6 (3)
O14W—Co3—O15W	87.98 (12)	C9—C10—C5	120.3 (3)
O13W—Co3—O15W	87.68 (11)	C9—C10—H10	119.8
O17W—Co3—O18W	93.78 (11)	C5—C10—H10	119.8
O14W—Co3—O18W	96.96 (11)	C16—C11—C12	120.7 (4)
O13W—Co3—O18W	87.21 (11)	C16—C11—N2	122.2 (3)
O15W—Co3—O18W	88.44 (11)	C12—C11—N2	117.1 (3)
O17W—Co3—O16W	84.57 (11)	C13—C12—C11	119.2 (4)
O14W—Co3—O16W	88.13 (11)	C13—C12—H12	120.4
O13W—Co3—O16W	87.83 (11)	C11—C12—H12	120.4
O15W—Co3—O16W	93.24 (11)	C14—C13—C12	120.9 (4)
O18W—Co3—O16W	174.70 (11)	C14—C13—H13	119.6
O2—S1—O3	113.05 (15)	C12—C13—H13	119.6
O2—S1—O1	111.34 (15)	C13—C14—C15	119.4 (4)
O3—S1—O1	111.08 (15)	C13—C14—H14	120.3
O2—S1—C7	108.61 (16)	C15—C14—H14	120.3
O3—S1—C7	107.80 (16)	C14—C15—C16	120.9 (4)
O1—S1—C7	104.52 (16)	C14—C15—H15	119.6
O5—S2—O6	112.94 (16)	C16—C15—H15	119.6
O5—S2—O4	111.83 (17)	C11—C16—C15	118.9 (4)
O6—S2—O4	111.87 (16)	C11—C16—H16	120.6
O5—S2—C9	107.29 (17)	C15—C16—H16	120.6
O6—S2—C9	105.89 (17)	N3—C17—C18	122.5 (3)
O4—S2—C9	106.52 (16)	N3—C17—C22	116.8 (3)
O9—S3—O8	113.44 (15)	C18—C17—C22	120.2 (3)
O9—S3—O10	112.07 (15)	O14—C18—C19	120.7 (3)
O8—S3—O10	109.83 (15)	O14—C18—C17	121.0 (3)
O9—S3—C23	106.34 (16)	C19—C18—C17	118.2 (3)
O8—S3—C23	110.14 (16)	C20—C19—C18	121.1 (4)
O10—S3—C23	104.58 (16)	C20—C19—H19	119.5
O13—S4—O12	112.53 (16)	C18—C19—H19	119.5
O13—S4—O11	112.68 (17)	C19—C20—C21	122.2 (4)
O12—S4—O11	111.26 (16)	C19—C20—H20	118.9
O13—S4—C25	107.35 (16)	C21—C20—H20	118.9
O12—S4—C25	106.12 (17)	C26—C21—C22	120.8 (3)
O11—S4—C25	106.41 (16)	C26—C21—C20	118.8 (3)
O16—S5—O15	114.18 (16)	C22—C21—C20	120.4 (3)
O16—S5—O17	111.30 (15)	C21—C22—C23	116.8 (3)
O15—S5—O17	111.24 (15)	C21—C22—C17	117.4 (3)
O16—S5—C39	107.52 (16)	C23—C22—C17	125.9 (3)
O15—S5—C39	107.18 (16)	C24—C23—C22	120.9 (3)
O17—S5—C39	104.82 (16)	C24—C23—S3	113.6 (3)
O20—S6—O19	112.47 (17)	C22—C23—S3	124.9 (3)
O20—S6—O18	112.27 (16)	C23—C24—C25	120.5 (3)
O19—S6—O18	111.72 (17)	C23—C24—H24	119.7
O20—S6—C41	107.48 (17)	C25—C24—H24	119.7
O19—S6—C41	105.72 (17)	C26—C25—C24	119.9 (3)
O18—S6—C41	106.70 (17)	C26—C25—S4	119.8 (3)
Co1—O1W—H1W	126 (2)	C24—C25—S4	120.3 (3)
Co1—O1W—H2W	123 (3)	C25—C26—C21	120.5 (3)
H1W—O1W—H2W	104 (2)	C25—C26—H26	119.7
Co1—O2W—H3W	120 (3)	C21—C26—H26	119.7
Co1—O2W—H4W	115 (3)	C32—C27—C28	120.9 (4)
H3W—O2W—H4W	101 (2)	C32—C27—N4	121.6 (3)
Co1—O3W—H5W	125 (3)	C28—C27—N4	117.5 (3)
Co1—O3W—H6W	125 (3)	C29—C28—C27	118.7 (4)
H5W—O3W—H6W	104 (3)	C29—C28—H28	120.6
Co1—O4W—H7W	107 (3)	C27—C28—H28	120.6
Co1—O4W—H8W	120 (3)	C30—C29—C28	120.9 (4)
H7W—O4W—H8W	104 (2)	C30—C29—H29	119.5
Co1—O5W—H9W	115 (3)	C28—C29—H29	119.5
Co1—O5W—H10W	117 (3)	C29—C30—C31	119.5 (4)
H9W—O5W—H10W	99 (2)	C29—C30—H30	120.3
Co1—O6W—H11W	126 (3)	C31—C30—H30	120.3
Co1—O6W—H12W	129 (3)	C30—C31—C32	120.7 (4)
H11W—O6W—H12W	101 (2)	C30—C31—H31	119.7
Co2—O7W—H13W	127 (3)	C32—C31—H31	119.7
Co2—O7W—H14W	121 (2)	C31—C32—C27	119.2 (4)
H13W—O7W—H14W	105 (3)	C31—C32—H32	120.4
Co2—O8W—H15W	113 (3)	C27—C32—H32	120.4
Co2—O8W—H16W	127 (3)	N5—C33—C34	122.4 (3)
H15W—O8W—H16W	104 (3)	N5—C33—C38	117.1 (3)
Co2—O9W—H17W	133 (3)	C34—C33—C38	120.0 (3)
Co2—O9W—H18W	121 (3)	O21—C34—C35	120.9 (3)
H17W—O9W—H18W	103 (2)	O21—C34—C33	120.5 (3)
Co2—O10W—H19W	120 (3)	C35—C34—C33	118.5 (3)
Co2—O10W—H20W	116 (3)	C36—C35—C34	120.7 (3)
H19W—O10W—H20W	102 (2)	C36—C35—H35	119.6
Co2—O11W—H21W	110 (3)	C34—C35—H35	119.6
Co2—O11W—H22W	121 (3)	C35—C36—C37	122.5 (4)
H21W—O11W—H22W	104 (2)	C35—C36—H36	118.7
Co2—O12W—H23W	129 (3)	C37—C36—H36	118.7
Co2—O12W—H24W	125 (2)	C42—C37—C38	121.8 (3)
H23W—O12W—H24W	102 (2)	C42—C37—C36	118.0 (3)
Co3—O13W—H25W	119 (3)	C38—C37—C36	120.2 (3)
Co3—O13W—H26W	124 (3)	C37—C38—C39	116.8 (3)
H25W—O13W—H26W	101 (2)	C37—C38—C33	117.5 (3)
Co3—O14W—H27W	125 (3)	C39—C38—C33	125.7 (3)
Co3—O14W—H28W	128 (3)	C40—C39—C38	119.9 (3)
H27W—O14W—H28W	103 (3)	C40—C39—S5	113.3 (3)
Co3—O15W—H29W	115 (3)	C38—C39—S5	126.7 (3)
Co3—O15W—H30W	127 (3)	C41—C40—C39	121.0 (3)
H29W—O15W—H30W	102 (2)	C41—C40—H40	119.5
Co3—O16W—H31W	120 (2)	C39—C40—H40	119.5
Co3—O16W—H32W	125 (3)	C42—C41—C40	120.4 (3)
H31W—O16W—H32W	100 (2)	C42—C41—S6	120.6 (3)
Co3—O17W—H33W	116 (3)	C40—C41—S6	119.0 (3)
Co3—O17W—H34W	112 (3)	C41—C42—C37	119.7 (3)
H33W—O17W—H34W	103 (3)	C41—C42—H42	120.2
Co3—O18W—H35W	120 (2)	C37—C42—H42	120.2
Co3—O18W—H36W	138 (3)	C44—C43—C48	121.3 (4)
H35W—O18W—H36W	102 (2)	C44—C43—N6	117.3 (3)
H37W—O19W—H38W	101 (2)	C48—C43—N6	121.4 (3)
H39W—O20W—H40W	99 (2)	C45—C44—C43	119.3 (4)
H41W—O21W—H42W	101 (2)	C45—C44—H44	120.4
H43W—O22W—H44W	101 (2)	C43—C44—H44	120.4
H45W—O23W—H46W	101 (2)	C46—C45—C44	120.5 (4)
H47W—O24W—H48W	99 (2)	C46—C45—H45	119.8
H49W—O25W—H50W	99 (2)	C44—C45—H45	119.8
H51W—O26W—H52W	100 (2)	C45—C46—C47	119.8 (4)
H53W—O27W—H54W	102 (3)	C45—C46—H46	120.1
H55W—O28W—H56W	100 (2)	C47—C46—H46	120.1
H57W—O29W—H58W	101 (2)	C46—C47—C48	120.7 (4)
H59W—O30W—H60W	103 (2)	C46—C47—H47	119.7
N2—N1—C1	118.8 (3)	C48—C47—H47	119.7
N1—N2—C11	121.3 (3)	C43—C48—C47	118.5 (4)
N1—N2—H1N	115 (3)	C43—C48—H48	120.8
C11—N2—H1N	123 (3)	C47—C48—H48	120.8
N4—N3—C17	119.3 (3)		
			
C1—N1—N2—C11	−175.7 (3)	O10—S3—C23—C24	16.1 (3)
C17—N3—N4—C27	175.7 (3)	O9—S3—C23—C22	−36.0 (3)
C33—N5—N6—C43	−175.4 (3)	O8—S3—C23—C22	87.3 (3)
N2—N1—C1—C2	6.9 (5)	O10—S3—C23—C22	−154.7 (3)
N2—N1—C1—C6	179.2 (3)	C22—C23—C24—C25	4.4 (5)
N1—C1—C2—O7	−11.4 (6)	S3—C23—C24—C25	−166.8 (3)
C6—C1—C2—O7	176.6 (3)	C23—C24—C25—C26	2.9 (5)
N1—C1—C2—C3	165.0 (3)	C23—C24—C25—S4	−177.6 (3)
C6—C1—C2—C3	−7.0 (5)	O13—S4—C25—C26	−92.0 (3)
O7—C2—C3—C4	177.4 (4)	O12—S4—C25—C26	28.5 (3)
C1—C2—C3—C4	0.9 (5)	O11—S4—C25—C26	147.1 (3)
C2—C3—C4—C5	2.6 (6)	O13—S4—C25—C24	88.4 (3)
C3—C4—C5—C10	179.7 (4)	O12—S4—C25—C24	−151.0 (3)
C3—C4—C5—C6	−0.1 (6)	O11—S4—C25—C24	−32.4 (3)
C10—C5—C6—C7	−6.8 (5)	C24—C25—C26—C21	−5.3 (5)
C4—C5—C6—C7	173.0 (3)	S4—C25—C26—C21	175.2 (3)
C10—C5—C6—C1	174.4 (3)	C22—C21—C26—C25	0.5 (5)
C4—C5—C6—C1	−5.8 (5)	C20—C21—C26—C25	179.9 (3)
N1—C1—C6—C7	18.2 (5)	N3—N4—C27—C32	1.4 (5)
C2—C1—C6—C7	−169.3 (3)	N3—N4—C27—C28	−179.3 (3)
N1—C1—C6—C5	−163.2 (3)	C32—C27—C28—C29	1.2 (6)
C2—C1—C6—C5	9.3 (5)	N4—C27—C28—C29	−178.1 (3)
C5—C6—C7—C8	8.1 (5)	C27—C28—C29—C30	0.4 (6)
C1—C6—C7—C8	−173.2 (3)	C28—C29—C30—C31	−1.4 (6)
C5—C6—C7—S1	−167.8 (3)	C29—C30—C31—C32	0.8 (6)
C1—C6—C7—S1	10.8 (5)	C30—C31—C32—C27	0.8 (6)
O2—S1—C7—C8	99.6 (3)	C28—C27—C32—C31	−1.8 (6)
O3—S1—C7—C8	−137.5 (3)	N4—C27—C32—C31	177.4 (3)
O1—S1—C7—C8	−19.3 (3)	N6—N5—C33—C34	7.6 (5)
O2—S1—C7—C6	−84.1 (3)	N6—N5—C33—C38	179.2 (3)
O3—S1—C7—C6	38.7 (4)	N5—C33—C34—O21	−12.4 (5)
O1—S1—C7—C6	156.9 (3)	C38—C33—C34—O21	176.2 (3)
C6—C7—C8—C9	−3.9 (5)	N5—C33—C34—C35	164.7 (3)
S1—C7—C8—C9	172.5 (3)	C38—C33—C34—C35	−6.7 (5)
C7—C8—C9—C10	−2.1 (5)	O21—C34—C35—C36	178.5 (4)
C7—C8—C9—S2	178.5 (3)	C33—C34—C35—C36	1.4 (5)
O5—S2—C9—C10	98.1 (3)	C34—C35—C36—C37	1.4 (6)
O6—S2—C9—C10	−22.7 (4)	C35—C36—C37—C42	−179.0 (4)
O4—S2—C9—C10	−142.0 (3)	C35—C36—C37—C38	1.1 (6)
O5—S2—C9—C8	−82.5 (3)	C42—C37—C38—C39	−5.2 (5)
O6—S2—C9—C8	156.7 (3)	C36—C37—C38—C39	174.7 (3)
O4—S2—C9—C8	37.4 (3)	C42—C37—C38—C33	173.9 (3)
C8—C9—C10—C5	3.5 (6)	C36—C37—C38—C33	−6.3 (5)
S2—C9—C10—C5	−177.1 (3)	N5—C33—C38—C37	−162.8 (3)
C6—C5—C10—C9	1.1 (6)	C34—C33—C38—C37	9.0 (5)
C4—C5—C10—C9	−178.7 (3)	N5—C33—C38—C39	16.1 (5)
N1—N2—C11—C16	−3.1 (5)	C34—C33—C38—C39	−172.0 (3)
N1—N2—C11—C12	177.8 (3)	C37—C38—C39—C40	6.7 (5)
C16—C11—C12—C13	−0.6 (6)	C33—C38—C39—C40	−172.3 (3)
N2—C11—C12—C13	178.5 (3)	C37—C38—C39—S5	−170.1 (3)
C11—C12—C13—C14	−0.5 (6)	C33—C38—C39—S5	11.0 (5)
C12—C13—C14—C15	0.9 (6)	O16—S5—C39—C40	100.8 (3)
C13—C14—C15—C16	−0.1 (6)	O15—S5—C39—C40	−136.0 (3)
C12—C11—C16—C15	1.3 (6)	O17—S5—C39—C40	−17.7 (3)
N2—C11—C16—C15	−177.7 (3)	O16—S5—C39—C38	−82.2 (3)
C14—C15—C16—C11	−1.0 (6)	O15—S5—C39—C38	40.9 (4)
N4—N3—C17—C18	−6.2 (5)	O17—S5—C39—C38	159.2 (3)
N4—N3—C17—C22	−178.3 (3)	C38—C39—C40—C41	−2.7 (5)
N3—C17—C18—O14	10.6 (5)	S5—C39—C40—C41	174.4 (3)
C22—C17—C18—O14	−177.5 (3)	C39—C40—C41—C42	−3.2 (6)
N3—C17—C18—C19	−166.6 (3)	C39—C40—C41—S6	176.6 (3)
C22—C17—C18—C19	5.3 (5)	O20—S6—C41—C42	−10.1 (4)
O14—C18—C19—C20	−176.2 (3)	O19—S6—C41—C42	110.3 (3)
C17—C18—C19—C20	1.0 (5)	O18—S6—C41—C42	−130.7 (3)
C18—C19—C20—C21	−3.2 (6)	O20—S6—C41—C40	170.1 (3)
C19—C20—C21—C26	179.7 (4)	O19—S6—C41—C40	−69.5 (3)
C19—C20—C21—C22	−0.9 (6)	O18—S6—C41—C40	49.5 (3)
C26—C21—C22—C23	6.4 (5)	C40—C41—C42—C37	4.8 (5)
C20—C21—C22—C23	−173.0 (3)	S6—C41—C42—C37	−175.0 (3)
C26—C21—C22—C17	−173.6 (3)	C38—C37—C42—C41	−0.5 (6)
C20—C21—C22—C17	7.0 (5)	C36—C37—C42—C41	179.7 (3)
N3—C17—C22—C21	163.2 (3)	N5—N6—C43—C44	179.6 (3)
C18—C17—C22—C21	−9.1 (5)	N5—N6—C43—C48	−2.2 (5)
N3—C17—C22—C23	−16.8 (5)	C48—C43—C44—C45	−0.5 (6)
C18—C17—C22—C23	170.9 (3)	N6—C43—C44—C45	177.7 (3)
C21—C22—C23—C24	−8.8 (5)	C43—C44—C45—C46	0.1 (6)
C17—C22—C23—C24	171.2 (3)	C44—C45—C46—C47	0.2 (6)
C21—C22—C23—S3	161.4 (3)	C45—C46—C47—C48	−0.2 (6)
C17—C22—C23—S3	−18.6 (5)	C44—C43—C48—C47	0.5 (6)
O9—S3—C23—C24	134.8 (3)	N6—C43—C48—C47	−177.6 (3)
O8—S3—C23—C24	−101.9 (3)	C46—C47—C48—C43	−0.1 (6)
